# Comparison of treatment plans between IMRT with MR-linac and VMAT for lung SABR

**DOI:** 10.1186/s13014-019-1314-0

**Published:** 2019-06-13

**Authors:** Jong Min Park, Hong-Gyun Wu, Hak Jae Kim, Chang Heon Choi, Jung-in Kim

**Affiliations:** 1 Department of Radiation Oncology, Seoul National University Hospital, Seoul, South Korea; 2 Institute of Radiation Medicine, Seoul National University Medical Research Center, Seoul, South Korea; 3 Biomedical Research Institute, Seoul National University Hospital, Seoul, South Korea; 4 Institute for Smart System, Robotics Research Laboratory for Extreme Environments, Advanced Institutes of Convergence Technology, Suwon, South Korea; 5 Department of Radiation Oncology, Seoul National University College of Medicine, Seoul, South Korea

**Keywords:** MR-IGRT, MR-linac, VMAT, Planning study, SABR

## Abstract

**Background:**

The aim of this study was to compare the plan quality of magnetic-resonance image-based intensity modulated radiation therapy (MRI-based-IMRT) with the MRIdian Linac system to that of volumetric modulated arc therapy (VMAT) with the TrueBeam STx system for lung stereotactic ablative radiotherapy (SABR).

**Methods:**

A total of 22 patients with tumors located in the lower lobe were retrospectively selected for the study. For each patient, both the MRI-based-IMRT and VMAT plans were generated using an identical CT image set and identical structures with the exception of the planning target volume (PTV). The PTVs of the MRI-based-IMRT were generated by adding an isotropic margin of 3 mm from the gross tumor volume, whereas those of VMAT were generated by adding an isotropic margin of 5 mm from the internal target volume. For both the MRI-based-IMRT and VMAT, the prescription doses to the PTVs were 60 Gy in four fractions.

**Results:**

The average PTV volume of the MRI-based-IMRT was approximately 4-times smaller than that of VMAT (*p* <  0.001). The maximum dose to the bronchi for the MRI-based-IMRT was smaller than that for the VMAT (20.4 Gy versus 24.2 Gy, *p* <  0.001). In addition, V_40Gy_ of the rib for the MRI-based-IMRT was smaller than that for the VMAT (1.8 cm^3^ versus 7.7 cm^3^, *p* = 0.008). However, the maximum doses to the skin and spinal cord for the MRI-based-IMRT (33.0 Gy and 14.5 Gy, respectively) were larger than those for the VMAT (27.8 Gy and 11.0 Gy, respectively) showing *p* values of less than 0.02. For the ipsilateral lung, the mean dose, V_20Gy_, V_10Gy_, and V_5Gy_ for the MRI-based-IMRT were smaller than those for the VMAT (all with *p* <  0.05). For the contralateral lung, V_5Gy_, V_10Gy_, D_1500cc_, and D_1000cc_ for the MRI-based-IMRT were larger than those for the VMAT (all with *p* <  0.05). The mean dose and V_50%_ of the whole body for the MRI-based-IMRT were smaller than those for the VMAT (0.9 Gy versus 1.2 Gy, and 78.7 cm^3^ versus 103.5 cm^3^, respectively, all at *p* <  0.001).

**Conclusions:**

The MRI-based-IMRT using the MRIdian Linac system could reduce doses to bronchi, rib, ipsilateral lung, and whole body compared to VMAT for lung SABR when the tumor was located in the lower lobe.

## Background

Since the release of the MRIdian® Linac system (ViewRay Inc., Oakwood Village, OH), linear accelerators with a magnetic resonance imaging system (MR-linac) have become clinically available. The MRIdian Linac system can generate 6 MV flattening filter free (FFF) beams with a maximum dose rate of 6 Gy/min using an S-band standing wave linear accelerator (linac) [1]. Therefore, the MRIdian Linac system generates treatment beams with higher penetrability and smaller penumbrae than those of the ViewRay® system (ViewRay Inc., Oakwood Village, OH), which is a previous model for magnetic resonance image-guided radiation therapy (MR-IGRT) that uses Co-60 radioisotopes to generate treatment beams [2, 3]. Although the MRIdian Linac system is equipped with a linac, 3D volumetric and 2D planar magnetic resonance images (MRI) can be acquired using a 0.35 T magnetic field, similar with the ViewRay system [1]. The mutual interference that occurs between the linac and MR imaging systems of the MRIdian Linac system were eliminated using patented technology from ViewRay, Inc. [1]. In addition to the successful integration of the linac and MR imaging systems, a notable feature of the MRIdian Linac system is a double-stacked and double-focused multi-leaf collimator (MLC) system that can project field sizes of 0.2 cm × 0.4 cm to 27.4 cm × 24.1 cm at the isoplane located 90 cm from the source [1]. Using this MLC system, the MRIdian Linac system can conduct step-and-shoot intensity modulated radiation therapy (IMRT) with fluences at a finer resolution than those of the ViewRay system, thereby potentially improving the quality of the treatment plan [4, 5].

One of the indications of MR-IGRT is lung cancer owing to the substantial respiratory motions of the lung tumors [6]. Because the MR-IGRT technique can be used in respiratory gated radiation therapy based on the application of real-time cine planar MR images during treatment without additional imaging doses, the margins of the lung target volumes can be significantly reduced, which results in substantially smaller doses to normal tissue [2]. This advantage can be maximized in stereotactic ablative radiotherapy (SABR) for lung cancer because the fractional doses of lung SABR are much larger than those of conventional fractionated radiation therapy and should be delivered to patients accurately for lung SABR [7]. Several previous studies have compared the dose distributions of MR-IGRT using the ViewRay system to those of linac-based IMRT or linac-based volumetric modulated arc therapy (VMAT) and reported that the plan quality of the ViewRay system is clinically acceptable but no better than that of linac-based IMRT and VMAT owing to the inferior beam quality of the Co-60 source and the large MLC leaf width of the ViewRay system [2, 3, 8–11]. Specifically for lung target volumes of smaller than 10 cm^3^, the plan quality of internal target volume (ITV) based VMAT is statistically significantly better than that of gross tumor volume (GTV) based IMRT using the ViewRay system despite the margin reduction capability of the ViewRay system [2].

Because the MRIdian Linac system overcomes the disadvantages of the ViewRay system, including the poor characteristics of the Co-60 source beams and the large MLC leaf width, the plan quality of the MRIdian Linac system might be better than that of a conventional IMRT or VMAT by virtue of its margin reduction capability. In contrast, the MRIdian Linac system can only generate coplanar step-and-shoot IMRT plans whose plan quality might be inferior to that of VMAT. It is therefore unclear whether the lung SABR plan quality of the MRIdian Lianc system is better than that of VMAT. Thus, in the present study, we compared lung SABR IMRT plans using the MRIdian Linac system based on the GTV to those of VMAT plans using TrueBeam STx™ (Varian Medical Systems, Palo Alto, CA) based on the ITV for a total of 22 patients with lung cancer.

## Methods

### Patient selection and simulation

After receiving approval from the institutional review board, a total of 22 patients treated for lung cancer with the SABR VMAT technique were retrospectively selected for this study. Because the respiratory motion of lung tumors located in the lower lobe is known to be large, we selected only patients with lung tumors located in the lower lobe [6]. All patients were scanned using the Brilliance CT Big Bore™ (Phillips, Cleveland, OH). Ten phase 4D CT images were acquired using the Real-time Position Management™ (RPM, Varian Medical Systems, Palo Alto, CA) system for each patient to define the ITV for VMAT planning. The slice thickness of the CT images was 2 mm. When acquiring the CT images, all patients were immobilized using the Body Pro-Lok™ (CIVICO, Orange City, IA) system to reduce the respiratory motion of the lung tumors.

### Treatment planning of VMAT using TrueBeam STx

The planning target volume (PTV) was defined by adding an isotropic margin of 5 mm from the ITV in the 80% phase CT image set [7]. The prescription dose to the PTV was 60 Gy in four fractions for all patients. VMAT plans were generated using a 6 MV FFF beam of TrueBeam STx with a High-Definition 120 MLC™ (Varian Medical Systems, Palo Alto, CA). For the planning, the Eclipse™ system (Varian Medical Systems, Palo Alto, CA) was used. To minimize the doses to the contralateral lung, two coplanar half arcs near the ipsilateral lung were used. One half arc was rotated from 180° to 0°, whereas the other half arc was rotated from 0° to 180°. All VMAT plans were optimized using the progressive resolution optimizer (PRO3, ver. 13, Varian Medical Systems, Palo Alto, CA). The dose-volume constraints during optimization were set according to the National Comprehensive Cancer Network Clinical Practice Guidelines in Oncology (NCCN Guidelines®, National Comprehensive Cancer Network, Fort Washington, PA). After optimization, the dose distributions were calculated using the Acuros® XB algorithm (AXB, ver. 13, Varian Medical Systems, Palo Alto, CA). The size of the dose calculation grid was 1 mm, which is the finest resolution available with the Eclipse system. After a dose calculation, all plans were normalized to cover 95% of the PTV volume with 100% of the prescription dose.

### Treatment planning of IMRT using MRIdian Linac system

Because this is a comparative planning study, CT images identical to those of VMAT plans were used for the IMRT planning with the MRIdian Linac system. The PTV was defined by adding an isotropic margin of 3 mm from the GTV in the 80% phase CT image set because the MRIdian Linac system is capable of anatomy-based gated radiation therapy. The PTV was used as the gating window during treatment, i.e., the treatment beams were delivered to a patient only when the GTV was inside the PTV. We added a 3-mm isotropic PTV margin empirically to consider the daily variations in lung tumor movements of the patients. The prescription dose to the PTV was identical to that of the VMAT plans, which was 60 Gy in the four fractions. Coplanar step-and-shoot IMRT plans were generated for each patient with a 6 MV FFF photon beam of the MRIdian Linac system using double-stacked and double-focused MLCs. For the planning, the treatment planning system (TPS) of the MRIdian Linac system which is the MRIdian system (ViewRay Inc., Oakwood Village, OH) was used. Eight or nine fields with various gantry angles were used for each patient according to the tumor locations. The isocenter was not moved laterally to avoid collisions between the patient body and bore of the MRIdian Linac system. All plans were optimized to meet the dose-volumetric constraints based on the same NCCN guidelines as used in the VMAT planning. The sequencing number of segments was set to 60 [1]. The IMRT efficiency was set to 3.00, which determines the smoothness of the fluence map [1]. The dose distributions were calculated using a dose calculation grid of 2 mm. When calculating the dose distributions, the number of histories per cm^2^ was set to 25,000. After the dose calculation, the calculated dose distributions were imported from the MRIdian system to the Eclipse system in order to calculate the dose-volumetric parameters and dose volume histograms (DVHs) [12]. All plans were normalized to cover 100% of the PTV volume with 99.99% of the prescription dose because the PTV of the MRIdian Linac system was generated without considering the internal target motion through respiration.

### Evaluation of the treatment plans

For each plan, the total monitor unit (MU) and calculated beam-on time in the TPS were acquired. For the IMRT plans using the MRIdian Linac system, the average number of segments for each plan was acquired.

For the PTVs, the maximum, minimum, and mean doses of the PTV and PTV volumes were calculated. The minimum doses to 2% volume of the PTV (D_2%_), D_5%_, D_95%_, and D_98%_ were also calculated. The *homogeneity index* (*HI*) was calculated as follows [13].

1 $$ \mathrm{Homogeneity}\ \mathrm{index}\ \left(\mathrm{HI}\right)=\frac{D_{2\%}-{D}_{98\%}}{mean\ dose} $$

For organs at risk (OARs), the maximum doses to the bronchi, heart, rib, skin, and spinal cord were calculated. The percent volume received at least 34.8 Gy (V_34.8Gy_) for the bronchi, V_30Gy_ for the esophagus, V_34Gy_ for the heart, V_40Gy_ for the rib, V_36Gy_ for the skin, and V_26Gy_ for the spinal cord were also calculated. For both the ipsilateral and contralateral lungs, V_20Gy_, V_10Gy_, V_5Gy_, D_1500cc_, D_1000cc_, and mean doses were calculated.

For whole body of a patient, mean dose to whole body as well as the percent volume received at least 50% of the prescription dose (V_50%_) were acquired.

A paired t-test was conducted to examine the statistical significances of the differences in the parameters between VMAT and IMRT using the MRIdian Linac system. We regarded differences with *p* values of less than 0.05 as statistically significant.

## Results

### Differences in the plan parameters

The plan parameters of both VMAT and IMRT using an MR-linac are shown in Table 1. The MU of the MRI-based IMRT was twice that of VMAT on average (8373 MU for MRI-based IMRT versus 4173 MU for VMAT, *p* <  0.001). The calculated beam-on time of the MRI-based IMRT was approximately 4-times larger than that of VMAT on average (18.4 min for MRI-based IMRT versus 4.8 min for VMAT, *p* <  0.001).

**Table 1:** Plan information

	VMAT	IMRT with MR-linac	*p*
Monitor unit (MU)	4173 ± 439	8373 ± 1582	*< 0.001*
Calculated beam on time (min)	4.8 ± 1.0	18.4 ± 2.2	*< 0.001*
Number of fields	2 ± 0	8 ± 1	*< 0.001*
Number of segments	N/A	54 ± 7	–

Note: *VMAT* volumetric modulated arc therapy, *IMRT* intensity modulated radiation therapy, *MR-linac* magnetic resonance image linear accelerator

### Dose-volumetric parameters of PTV

**Fig. 1:**
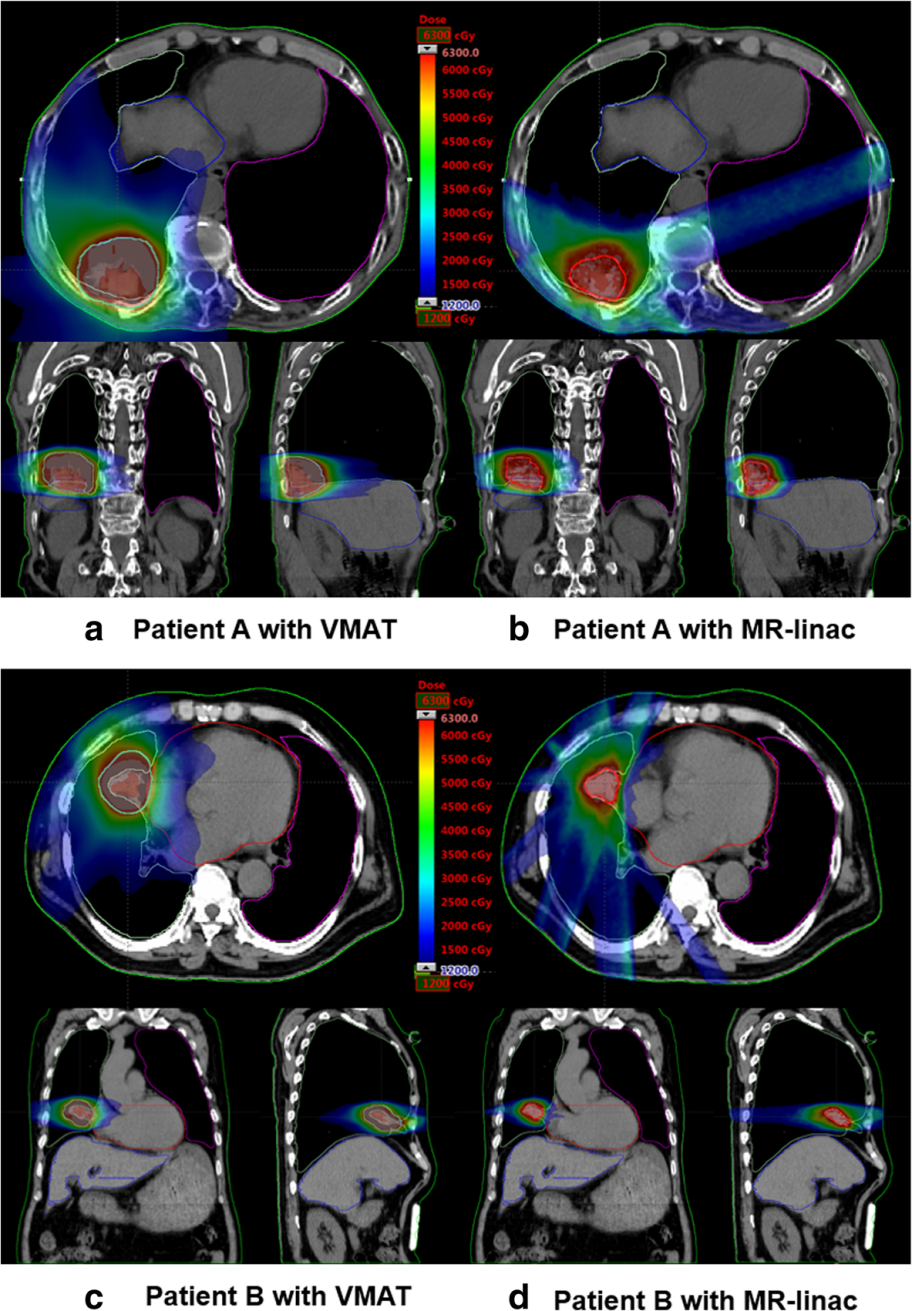
Representative patient dose distributions of volumetric modulated arc therapy and magnetic resonance image-based intensity modulated radiation therapy. Dose distributions in the axial, coronal, and sagittal planes of volumetric modulated arc therapy (VMAT) of representative patients, namely, patients A (**a**) and B (**c**), are shown. Those of magnetic resonance image-based intensity modulated radiation therapy (MRI-based IMRT) using a linear accelerator with a magnetic resonance imaging system are shown for patients A (**b**) and B (**d**). The planning target volume of VMAT were generated by adding an isotropic margin of 5 mm from the internal target volume, whereas those of MRI-based IMRT were generated by adding an isotropic margin of 3 mm from the gross tumor volume

**Fig. 2:**
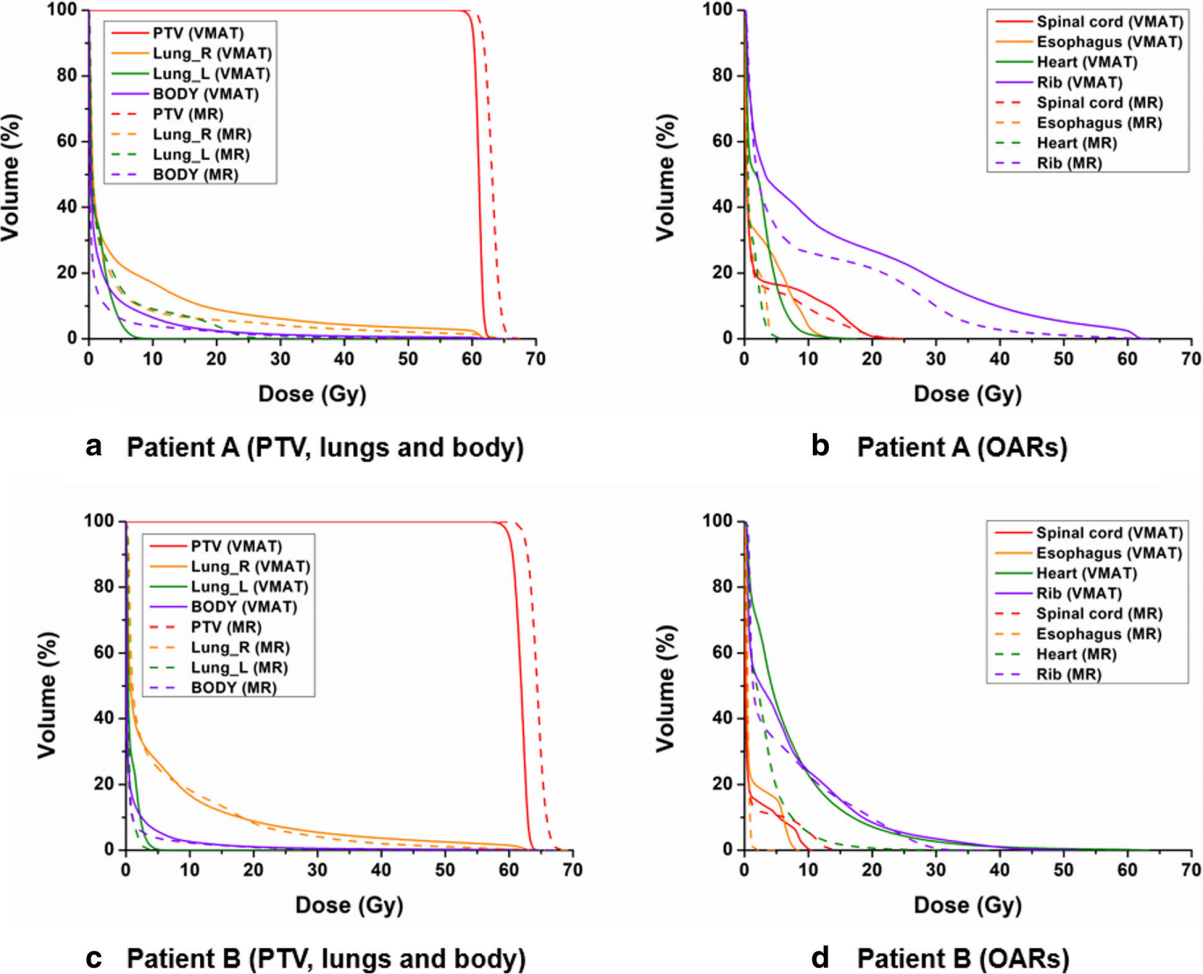
Representative dose-volume histograms of the target volume and organs at risk of volumetric modulated arc therapy and magnetic resonance image-based intensity modulated radiation therapy plans. Dose volume histograms of the planning target volume, both lungs, and whole body from the volumetric modulated arc therapy and magnetic resonance image-based intensity modulated radiation therapy plans of representative patients, namely, patients A (**a**) and B (**c**) are shown. Those of organs at risk of patients A (**b**) and B (**d**) are also shown

The dose-volumetric parameters of the PTVs of both the VMAT and MRI-based IMRT plans are shown in Table 2. The dose distributions of VMAT and MRI-based IMRT for the representative patients are shown in Fig. 1. The DVHs of the representative patients are shown in Fig. 2.

**Table 2:** Dose-volumetric parameters of the PTV

DV parameter	VMAT	IMRT with MR-linac	*p*
D_98%_ (Gy)	59.1 ± 0.3	60.3 ± 1.1	*< 0.001*
D_95%_ (Gy)	60.0 ± 0.0	60.7 ± 1.2	*0.015*
D_5%_ (Gy)	64.9 ± 1.3	63.8 ± 1.6	*0.026*
D_2%_ (Gy)	65.3 ± 1.4	64.2 ± 1.7	*0.035*
Minimum dose (Gy)	54.6 ± 1.4	58.7 ± 1.3	*< 0.001*
Maximum dose (Gy)	66.8 ± 1.3	63.8 ± 1.5	*< 0.001*
Mean dose (Gy)	63.0 ± 0.8	62.3 ± 1.4	*0.047*
PTV volume (cm^3^)	27.2 ± 23.5 (ITV + 5 mm)	6.3 ± 7.7 (GTV + 3 mm)	*< 0.001*
Homogeneity index	0.099 ± 0.023	0.062 ± 0.014	*< 0.001*

Note: *DV parameter* dose-volumetric parameter, *VMAT* volumetric modulated arc therapy, *IMRT* intensity modulated radiation therapy, *MR-linac* magnetic resonance image linear accelerator, *D*_*n%*_ dose received by at least *n*% volume of the planning target volume, *PTV* planning target volume, *ITV* internal target volume, *GTV* gross tumor volume

The PTV volume of the MRI-based IMRT is approximately 4-times smaller than that of VMAT on average (*p* <  0.001). The average values of D_98%_, D_95%_, and the minimum dose of MRI-based IMRT were larger than those of VMAT (all at *p* <  0.02) while the average values of D_5%_, D_2%_, and the maximum dose of MRI-based IMRT were smaller than those of VMAT (all at *p* <  0.04). Therefore, *HI* indicates that the dose homogeneity inside the PTV volume of MRI-based IMRT was better than that of VMAT (0.062 for MRI-based IMRT versus 0.099 for VMAT, *p* <  0.001).

### Dose-volumetric parameters of OARs

The dose-volumetric parameters of OARs are shown in Table 3.

**Table 3:** Dose-volumetric parameters of OARs and whole body

OAR	DV parameter	VMAT	IMRT with MR-linac	*p*
Bronchi	Maximum dose (Gy)	24.2 ± 21.7	20.4 ± 19.0	*< 0.001*
	V_34.8Gy_ (cm^3^)	0.5 ± 2.0	0.0 ± 0.1	0.223
Esophagus	V_30Gy_ (cm^3^)	0.0 ± 0.0	0.0 ± 0.0	–
Heart	Maximum dose (Gy)	20.2 ± 16.0	19.1 ± 13.7	0.087
	V_34Gy_ (cm^3^)	0.7 ± 2.4	0.0 ± 0.1	0.168
Ipsilateral lung	Mean dose (Gy)	5.7 ± 2.0	5.2 ± 2.1	*0.041*
	V_20Gy_ (cm^3^)	134.8 ± 81.4	116.8 ± 80.9	*0.025*
	V_10Gy_ (cm^3^)	275.3 ± 139.3	244.7 ± 140.2	*0.047*
	V_5Gy_ (cm^3^)	414.7 ± 182.0	358.1 ± 172.9	*< 0.001*
	D_1500cc_ (Gy)	0.13 ± 0.25	0.24 ± 0.37	*0.001*
	D_1000cc_ (Gy)	0.61 ± 1.15	0.76 ± 0.94	*0.012*
Contralateral lung	Mean dose (Gy)	0.9 ± 0.4	1.0 ± 0.6	0.210
	V_20Gy_ (cm^3^)	0.0 ± 0.0	6.6 ± 20.1	0.146
	V_10Gy_ (cm^3^)	0.5 ± 1.6	23.1 ± 44.3	*0.029*
	V_5Gy_ (cm^3^)	23.4 ± 37.1	49.8 ± 72.1	*0.049*
	D_1500cc_ (Gy)	0.06 ± 0.10	0.11 ± 0.14	*0.002*
	D_1000cc_ (Gy)	0.21 ± 0.28	0.28 ± 0.17	*0.046*
Rib	Maximum dose (Gy)	47.2 ± 16.5	43.0 ± 12.9	*0.032*
	V_40Gy_ (cm^3^)	7.7 ± 13.0	1.8 ± 3.9	*0.008*
Skin	Maximum dose (Gy)	27.8 ± 9.4	33.0 ± 4.4	*0.018*
	V_36Gy_ (cm^3^)	0.1 ± 0.4	0.1 ± 0.2	0.167
Spinal cord	Maximum dose (Gy)	11.0 ± 5.1	14.5 ± 5.6	*0.002*
	V_26Gy_ (cm^3^)	0.0 ± 0.0	0.0 ± 0.0	–
Whole body	Mean dose (Gy)	1.2 ± 0.5	0.9 ± 0.3	*< 0.001*
	V_50%_ (cm^3^)	103.5 ± 82.8	78.7 ± 62.9	*< 0.001*

Note: *OAR* organ at risk, *DV parameter* dose-volumetric parameter, *VMAT* volumetric modulated arc therapy, *IMRT* intensity modulated radiation therapy, *MR-linac* magnetic resonance image linear accelerator, *V*_*nGy*_ percent volume receiving *n* Gy, *D*_*ncc*_ dose received by at least *n* cc volume of the planning target volume, *V*_*n%*_ percent volume receiving *n*% of the prescription dose

The maximum dose to the bronchi of MRI-based IMRT was smaller than that of VMAT with a statistical significance (20.4 Gy for MRI-based IMRT versus 24.2 Gy for VMAT, *p* <  0.001). Both the maximum dose and V_34Gy_ for the heart were smaller in the MRI-based IMRT plans than in the VMAT plans; however, the differences were not statistically significant (all at *p* > 0.05). The maximum dose and V_40Gy_ of the rib of MRI-based IMRT (43.0 Gy and 1.8 cm^3^, respectively) were smaller than those of VMAT (47.2 Gy and 7.7 cm^3^, respectively) showing *p* values less than 0.04, whereas the maximum doses to the skin and spinal cord of MRI-based IMRT (33.0 and 14.5 Gy, respectively) were larger than those of VMAT (27.8 and 11.0 Gy, respectively) showing *p* values of less than 0.02.

For the ipsilateral lung, the mean dose, V_20Gy_, V_10Gy_, and V_5Gy_ of the MRI-based IMRT were smaller than those of VMAT (all at *p* <  0.05), whereas D_1500cc_ and D_1000cc_ of the MRI-based IMRT were larger than those of VMAT (all at *p* <  0.02). For the contralateral lung, V_10Gy_, V_5Gy_, D_1500cc_, and D_1000cc_ of the MRI-based IMRT were larger than those of VMAT with a statistical significance (all at *p* <  0.05).

### Dose-volumetric parameters of the whole body

The dose-volumetric parameters of the whole body are shown in Table 3. The mean dose and V_50%_ consistently indicated that the irradiation of normal tissue of MRI-based IMRT was lower than that of VMAT with a statistical significance (all at *p* <  0.001).

## Discussion

In the present study, we compared GTV-based IMRT plans generated with the MR-linac to the ITV-based VMAT plans for lung SABR. We retrospectively selected patients with tumors located at lower lobes of the lungs, of which the respiratory motions were large [6]. Therefore, the PTV sizes of VMAT, including margins compensating for the respiratory motion of the lung tumors, were much larger than those of MRI-based IMRT (approximately 4.3-times larger), which did not include the margins for respiratory motion. By doing so, we were able to maximize the advantage of MRI-based IMRT, which is internal anatomy-based gated radiotherapy with real-time cine MR images. Despite the smaller PTV sizes of MR-IGRT than those of conventional ITV-based radiotherapy, a previous study demonstrated that the lung SABR plan quality of the ViewRay system was not better than that of VMAT owing to the poor characteristics of Co-60 beams (large penumbrae and low penetrability) and the large MLC width of the ViewRay system [2]. Specifically for target volumes of smaller than 10 cm^3^, no benefits were observed when utilizing the ViewRay system for lung SABR as compared to VMAT [2]. However, in the present study, the MR-IGRT plans with the MR-linac demonstrated superiority to the VMAT plans even for target volumes of smaller than 10 cm^3^, showing less irradiation of normal tissue with intermediate and high doses than in the VMAT plans. As the results show, the volumes of body irradiated by 50% of the prescription dose of MRI-based IMRT were approximately 76% that of VMAT. In addition, the MRI-based IMRT was generally able to reduce the dose-volumetric parameters of the bronchi, rib, heart, and ipsilateral lung as compared to VMAT. This better plan quality was attributed to the margin reduction capability of MRI-based IMRT combined with the improved photon beam characteristics generated using the linac and small leaf widths of the MLCs.

Although most of the clinically relevant dose-volumetric parameters of MRI-based IMRT were better than those of VMAT, this was not always the case. Regarding the D_1500cc_ and D_1000cc_ of the ipsilateral lung, and all dose-volumetric parameters of the contralateral lung examined in this study, the maximum dose to the skin, and the maximum dose to the spinal cord from the MR-IGRT plans were worse than those of the VMAT plans. This might be attributed to the different characteristics of the IMRT and VMAT technique as well as the different beam arrangements of their plans, as described in the present study [13, 14]. For D_1500cc_ and D_1000cc_ of the ipsilateral lung, the average differences between MRI-based IMRT and VMAT were less than only 0.15 Gy, which is insignificant. For the contralateral lung, VMAT used half arcs proximal to the ipsilateral lung, and therefore no beams were delivered through the contralateral lung. In contrast, for MRI-based IMRT plans, beams were occasionally delivered to the target volume through the contralateral lung to acquire the acceptable target conformity, as shown in Fig. 1 (b). This resulted in relatively higher dose-volumetric parameters of the contralateral lung in the MRI-based IMRT plans than in the VMAT plans. In the case of skin, because the VMAT technique delivers beams through arcs, i.e., numerous beams in various directions are utilized, the doses can be distributed all over the skin but not concentrated within specific regions [13]. This can reduce the maximum doses to the skin for VMAT plans. In the case of the spinal cord, because MRI-based IMRT occasionally uses a beam through the spinal cord according to the target volume location, as shown in Fig. 1 (d), this could increase the maximum dose to the spinal cord.

The required MUs and beam-on times of MRI-based IMRT were much larger than those of VMAT. According to a simple calculation, a beam-on time of approximately 4-times longer on average was required for MRI-based IMRT as compared to VMAT, which increases the patient discomfort. In addition, the respiratory gated beam delivery of MRI-based IMRT would increase the treatment time. However, the patient setup of MRI-based IMRT is relatively simpler than that of VMAT for lung SABR because rigorous immobilization devices used to suppress the respiratory motion of the patient to reduce the ITV margins are not necessarily applied to a patient in the case of MRI-based IMRT. This simple setup could increase the degree of patient comfort. Therefore, MRI-based IMRT has both advantages and disadvantages in terms of patient comfort. Besides the patient comfort issue, for the long beam-on time of MRI-based IMRT, there might be a concern regarding an inaccurate treatment when the treatment time is long. However, a previous study reported that a long treatment time is not correlated with an inaccurate treatment for lung SABR [15]. Moreover, because the treatment beams of MRI-based IMRT are delivered to patients when monitoring their internal anatomy motion in real time, the long treatment time of MRI-based IMRT will not hamper the treatment accuracy.

Several studies comparing MRI-based IMRT using the ViewRay system to linac-based IMRT or VMAT for lung SABR have reported that the former are clinically acceptable but generally worse than those of linac-based IMRT or VMAT [2, 3, 8–11]. However, no studies have yet been conducted to compare MRI-based IMRT plans using the MRIdian Linac system to VMAT plans for lung SABR. In the present study, we conducted a planning study comparing MRI-based IMRT plans using the MRIdian Linac system to VMAT plans for lung SABR when the tumors were located in the lower lob. We presented that the MRI-based IMRT could reduce doses to bronchi, rib, ipsilateral lung, and whole body compared to VMAT and this was attributed to the combination of 6 MV FFF photon beams generated from the MR-linac, a double-stacked and double-focus MLC system with small leaf widths, and the margin reduction capability using a real-time anatomy-based gating technique.

## Conclusions

In the present study, we identified the MRI-based IMRT with the MRIdian Linac system could improve the dose homogeneity inside the target volume as well as reduce doses to bronchi, rib, ipsilateral lung, and whole body compared to VMAT for lung SABR when the tumors were located in the lower lobe. In addition, MRI-based IMRT with the MRIdian Linac system is capable of internal anatomy-based gated radiotherapy by monitoring real-time tumor motion, which guarantees an accurate treatment, but currently cannot be conducted using the VMAT technique. Therefore, it seems appropriate to utilize MRI-based IMRT for lung SABR when the tumors were located in the lower lobe.

## Abbreviations

AXB: Acuros XB
DVH: Dose volume histogram
FFF: Flattening filter free
GTV: Gross tumor volume
HI: Homogeneity index
IMRT: Intensity modulated radiation therapy
ITV: Internal target volume
linac: Linear accelerator
MLC: Multi-leaf collimator
MRI: Magnetic resonance image
MR-IGRT: Magnetic resonance image-guided radiation therapy
MR-linac: Linear accelerator with magnetic resonance imaging system
MU: Monitor unit
NCCN: National comprehensive cancer network
OAR: Organ at risk
PRO3: Progressive resolution optimizer 3
PTV: Planning target volume
RPM: Real-time Position Management
SABR: Stereotactic ablative radiotherapy
TPS: Treatment planning system
VMAT: Volumetric modulated arc therapy
